# The OsWRKY15‐OsSUT3 Regulatory Module Enhances Rice Pollen Fertility by Mediating Sucrose Allocation to Anthers

**DOI:** 10.1111/pbi.70738

**Published:** 2026-08-04

**Authors:** Qiuping Li, Chunlong Zhang, Shuaibing Wang, Jin Chen, Yandong Sun, Wenqing Ma, Yu Yong, Qinghui Yang, Lijuan Chen, Jiancheng Wen, Dandan Li

**Affiliations:** ^1^ Rice Research Institute, Yunnan Agricultural University Kunming China; ^2^ College of Agronomy and Biotechnology Yunnan Agricultural University Kunming China; ^3^ School of Agronomy and Life Sciences, Zhaotong University Zhaotong China; ^4^ School of Chemistry and Environmental Engineering Yuxi Normal University Yuxi China

**Keywords:** *OsSUT3*, OsWRKY15, pollen fertility, rice, starch accumulation, transcriptional regulation

## Abstract

Sucrose transport and starch accumulation are crucial carbohydrate metabolic processes in rice, playing essential roles during late pollen development to ensure pollen maturation, fertility and high grain production. However, the molecular mechanisms regulating sucrose transport and starch accumulation during rice pollen development remain elusive. In this study, we bred and characterized a semi‐fertile rice pollen variant, *sfp10*, which is derived from highland terraced red rice from Yunnan, China. This variant showed insufficient starch accumulation in pollen, reduced pollen fertility and decreased seed setting compared to the wild type. Using map‐based cloning, CRISPR/Cas9 mutagenesis and gene complementation, the *OsSUT3* gene, which causes the *sfp10* variant, was shown to encode a sucrose transporter (SUT) that transports sucrose into pollen for starch synthesis, ensuring pollen viability and fertility. Additionally, transcriptome analysis identified OsWRKY15, a WRKY transcription factor that exhibits a highly similar spatiotemporal expression pattern to *OsSUT3* in late‐developing panicles. Yeast one‐hybrid, EMSA, dual‐luciferase reporter and ChIP‐seq assays demonstrated that OsWRKY15 directly binds to the promoter region of *OsSUT3* and upregulates its expression. Mutation of *OsWRKY15* significantly reduced *OsSUT3* expression and impaired pollen fertility and seed setting and these defects were further exacerbated in the *wrky15/OsSUT3‐KO* double‐knockout lines, while *OsSUT3* overexpression in the *wrky15* background significantly rescued pollen fertility and seed setting. These findings demonstrate that the OsWRKY15‐OsSUT3 regulatory module is essential for sucrose transport and starch accumulation during late pollen development in rice, providing new insights into the transcriptional regulation of carbon allocation to anthers for rice fertility and grain production.

## 1 Introduction

In flowering plants, reproduction depends on pollen grain development and maturation (Ma 2005). The success of pollen‐stigma interactions and fertilization is influenced by environmental factors and, more critically, the intrinsic fertility and viability of the pollen grains themselves (McCormick 2013; Zou et al. 2022). As the primary site for pollen formation and development, the anther undergoes a precise and complex developmental process that is divided into 14 stages based on histological features (Zhang et al. 2011). Stages 12–14 represent pollen grain maturation and release, during which sugar molecules that are transported and stored in pollen cells for synthesizing starch, which is accumulated until the pollen grains are filled (He et al. 2021; Zhou et al. 2022; Wang, Deng, et al. 2023). Pollen cells with normal fertility require the high accumulation of starch, which is synthesized from monosaccharides (such as glucose and fructose) present in the pollen cytoplasm and disaccharides (such as sucrose) stored in pollen cell vacuoles by starch synthase (Baker and Baker 1979; Lee et al. 2016; Qu et al. 2021). As a major energy source for pollen germination after fertilization, starch is essential for maintaining pollen fertility and viability. Extensive research has been conducted on the specification and differentiation of microspores in the early stages of anther development (Nguyen et al. 2016; Liu et al. 2017; Tao et al. 2019), but little is known about the starch accumulation and filling processes, which are critical steps in male fertility that occur in the later stages.

Sucrose plays a central role in coordinating pollen maturation by supplying the substrates required for starch synthesis (Hackel et al. 2006; Wang et al. 2022), and sucrose uptake into the cytoplasm of pollen cells requires functional SUT proteins, which are membrane‐localized sucrose‐H^+^ symporters possessing 12 transmembrane domains (Sauer 2007; Li et al. 2022; Sun et al. 2023). However, only a few genes have been revealed as being involved in sucrose transport into pollen cells, and the regulatory mechanism for SUT genes is still not entirely clear. *AtSUC1* in *Arabidopsis* (Sivitz et al. 2008) and *OsSUT1* in rice (Hirose et al. 2010) are essential for providing energy via sucrose uptake during pollen germination on the stigma. Silencing *NtSUT3* in tobacco (Lemoine et al. 1999) and *SlSUT2* in tomato (Hansch et al. 2020) impairs carbohydrate uptake, alters the expression patterns of sucrose synthases and decreases pollen fertility and the pollen germination rate, but starch accumulation during pollen development is not affected by the disruption of these SUTs, suggesting that the mechanism underlying sugar transport for starch biosynthesis is still unknown. Previous studies have shown that SUTs are specifically expressed in pollen and play a role in pollen starch synthesis (Sun et al. 2019; Liu et al. 2021; Li, Zhang, Wen, et al. 2023). For example, in cucumber ( *Cucumis sativus* ), the downregulation of *CsSUT1* leads to male sterility by reducing the sugar and starch contents of pollen (Sun et al. 2019). In rice, a 31‐bp insertion/deletion (InDel) in the 5′ untranslated region (05′UTR) of *OsSUT3*, which is expressed in pollen, significantly influences pollen starch accumulation, pollen fertility and the seed setting rate (Li, Zhang, Wen, et al. 2023). These observations indicate a strong correlation between starch accumulation in pollen grains and SUT gene expression; however, the molecular mechanisms underlying starch accumulation in pollen remain unclear.

Starch granule accumulation and carbohydrate metabolism in pollen are also tightly regulated by multiple transcription factors (TFs). WRKY, one of the largest TF families, modulates many plant processes and is critical for pollen development and microspore maturation (Guan et al. 2014; Chen et al. 2018; Wang, Chen, et al. 2023). The defining feature of WRKYs is their DNA‐binding domain, which consists of nearly 60 amino acids and is specifically responsible for binding to the W‐box (C/T)TGAC(C/T) in the promoter region (Rushton et al. 2010). In *Arabidopsis*, TFs WRKY2 and WRKY34 regulate the expression of their target gene, *GPT1*, which encodes a plastid membrane‐localized transporter that imports glucose‐6‐phosphate (Glc6P) for starch and/or fatty acid biosynthesis in mature pollen, influencing pollen fitness and successful reproduction (Zheng, Deng, et al. 2018). GhWRKY22 in cotton modulates the expression of JAZ genes by directly binding to their promoters, regulating anther/pollen development (Wang et al. 2019). In rice, OsWRKY37 directly binds to the upstream promoter regions of *OsCOPT6* (copper transporter) and *OsYSL16* (yellow stripe‐like protein) and positively activates their expression, affecting pollen development, decreasing fertility and reducing grain yield under Cu‐deficient conditions (Ji et al. 2024). Despite WRKYs being crucial components of regulatory pathways in plant pollen development, there have been few reports regarding their regulatory roles in pollen sucrose transport.

In our previous research, we developed and characterized a semi‐fertile pollen variant, *sfp10*, by backcrossing natural semi‐fertile pollen variant *LSM* with wild‐type rice variety 93–11 for four generations. Compared to 93–11, *sfp10* had significantly reduced pollen fertility and a lower seed setting rate but no differences in other major agronomic traits. The candidate gene underlying this phenotype was preliminarily mapped to a 398‐kb region on chromosome 10 (Li, Zhang, Yang, et al. 2023). However, the causal gene responsible for the *sfp10* phenotype has not been cloned or functionally validated.

In this study, we expanded the mapping population and narrowed the candidate region to identify and validate *OsSUT3* as the causal gene responsible for the semi‐fertile pollen phenotype of *sfp10*. To further determine how OsSUT3 regulates sucrose transport and starch accumulation during late pollen development in rice, we analysed fertility‐related phenotypes, expression patterns and the sugar and starch contents in *OsSUT3* knockout, complementation and overexpression lines. Finally, we identified OsWRKY15 as an upstream regulator of *OsSUT3* through in vitro and in vivo promoter‐binding assays, and further validated the OsWRKY15‐OsSUT3 regulatory module using diverse transgenic lines. In conclusion, our findings reveal that *OsSUT3* plays a crucial role in sucrose uptake and starch accumulation in pollen during the late stages of rice anther development. Moreover, our research further clarifies that the transcription factor OsWRKY15 regulates the expression of *OsSUT3*, thereby controlling sucrose import into pollen and consequently influencing starch synthesis and accumulation. The discovery of the OsWRKY15‐OsSUT3 regulatory module deepens our mechanistic understanding of the pollen development process and offers new targets for fertility modulation in plants.

## 2 Results

### 2.1 Fine‐Mapping and Candidate Gene Identification in *sfp10*

Based on the previously reported 398‐kb interval of *sfp10*, we increased the number of F_2_ recombinant individuals originating from a cross between 93 and 11 and *sfp10* from 800 to 1285, and then performed fine mapping using InDel markers (Table S1) within the candidate interval. The candidate region was narrowed to a 141‐kb interval between InDel markers M44 and M17 (Figure 1a). Based on comparison with reference genome IRGSP‐1.0 (https://rapdb.dna.affrc.go.jp), we identified five candidate genes within this region (Figure 1a). A 267‐bp InDel variation, encompassing 77‐bp at the 3′‐end of the first exon and 190‐bp at the 5′‐end of the first intron, was identified in the *Os10g0404500* (*OsSUT3*) gene based on sequencing analysis of 93–11 and *sfp10* (Figures 1b and S1a,b). There were no differences in the other four candidate genes between 93 and 11 and *sfp10* (Figure S1a). Therefore, candidate gene *Os10g0404500* (*OsSUT3*) was considered responsible for the *sfp10* variation.

**FIGURE 1:**
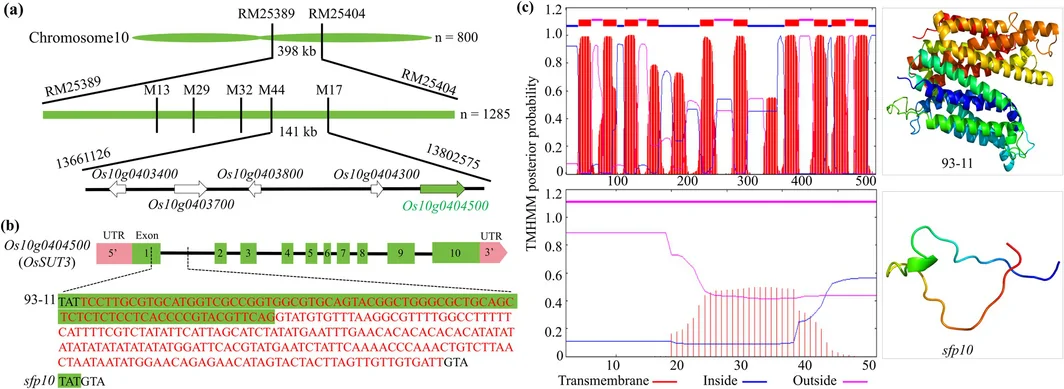
Map‐based cloning of the gene responsible for the *sfp10* variant. (a) Fine localization of *sfp10* in an F_2_ population constructed by crossing *sfp10* and 93–11. (b) Gene structure of *Os10g0404500*; red bases represent the 267‐bp deletion mutation spanning the first exon and the first intron, and the green background shows the deletion in the exon region. (c) Transmembrane domains (https://services.healthtech.dtu.dk/services/TMHMM‐2.0/) and Swiss‐Model (http://swissmodel.expasy.org) predictions of the OsSUT3 protein structure in 93–11 and *sfp10*.

To determine the effect of the 267‐bp InDel on protein function, we analysed the amino acid sequence of *OsSUT3* in 93–11 and *sfp10*. A 77‐bp deletion in the first exon of *sfp10 OsSUT3* caused a frameshift variation, leading to a premature stop codon. Thus, *OsSUT3* encoded a full‐length protein of 506 amino acids in 93–11, whereas it was predicted to encode a truncated protein of only 57 amino acids in *sfp10* (Figure S1c). Additionally, based on the TMHMM posterior probability, the topology model of the membrane protein predicted 12 transmembrane domains (TDs) in the OsSUT3 protein of 93–11. However, due to the 267‐bp deletion, no TDs were detected in *sfp10*, and this change was expected to induce significant structural variations in the OsSUT3 protein (Figure 1c). These results suggest that OsSUT3 malfunctions in *sfp10*.

### 2.2 *OsSUT3* Positively Regulates Pollen Fertility and the Seed Setting Rate

To determine whether *OsSUT3* serves as the causal gene of the *sfp10* variant, we generated knockout lines (KO‐1 and KO‐2) in the 93–11 background using the CRISPR/Cas9 system (Figure S2a), overexpression lines (OE‐1 and OE‐2) in which the *OsSUT3* gene of 93–11 was driven by the 35S promoter, and complementation lines (Com‐1 and Com‐2) in the *sfp10* background using an *OsSUT3* overexpression vector (Figure 2a). Agronomic trait observations showed no significant differences in plant height, number of tillers, flag leaf dimensions, or 1000‐grain weight among the rice lines used in this study (Figure S2b–e,g).

**FIGURE 2:**
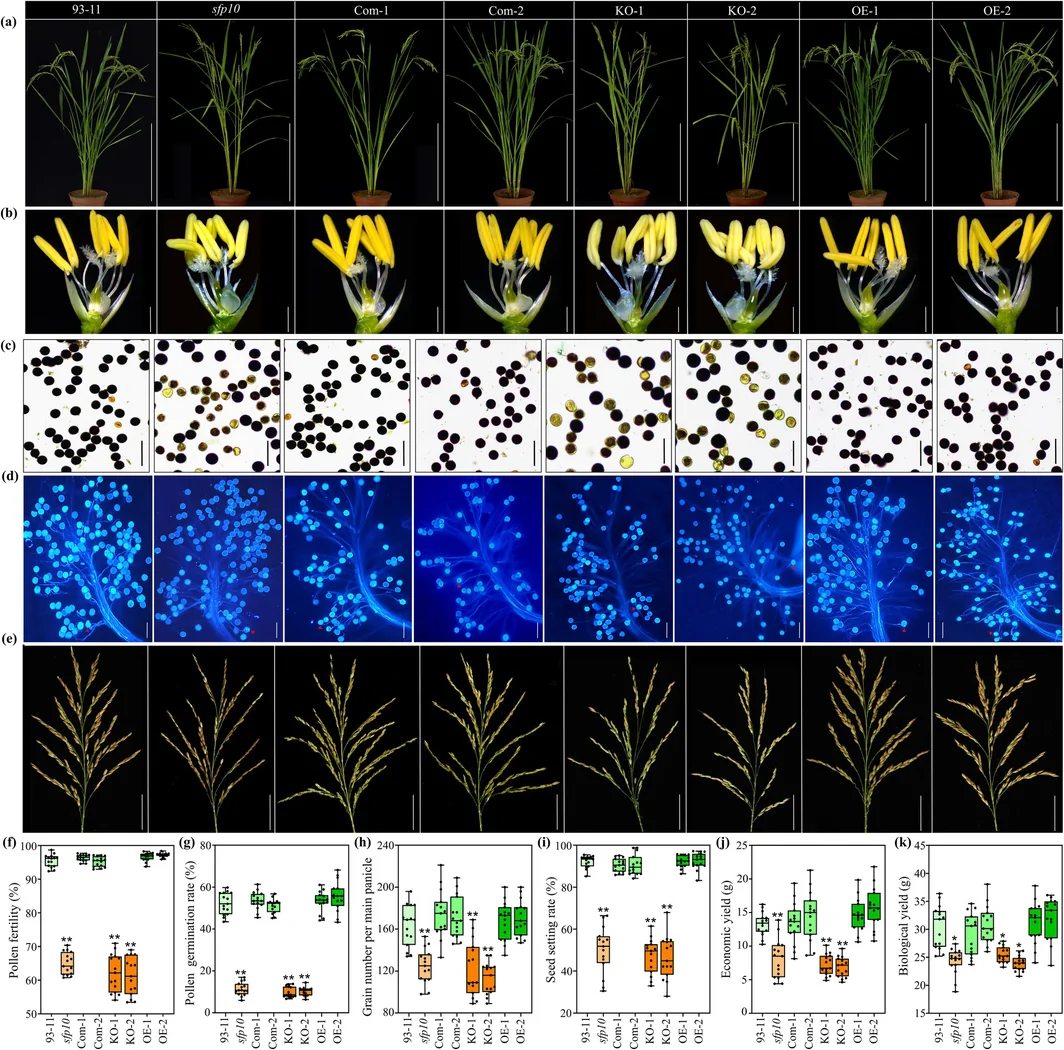
*OsSUT3* influences pollen fertility and seed setting in rice. (a) Phenotypes of whole rice plants of 93–11, *sfp10*, *OsSUT3* complementation (Com‐1, Com‐2), *OsSUT3* knockout (KO‐1, KO‐2), and *OsSUT3* overexpression (OE‐1, OE‐2) lines. Bar = 0.5 m. (b) Comparison of anther morphology at Stage 12 among 93–11, *sfp10*, Com, KO, and OE. Bar = 1 mm. (c) I_2_‐KI staining of pollen grains of 93–11, *sfp10*, Com, KO, and OE at Stage 12. Bar = 100 μm. (d) Pollen germination on the stigma of 93–11, *sfp10*, Com, KO, and OE after 0.5 h. Red triangles denote pollen tubes. Bar = 200 μm. (e) Phenotypes of the main culm panicles of 93–11, *sfp10*, Com, KO, and OE at the mature stage. Bar = 5 cm. (f,g) Comparison of pollen fertility (f) and pollen germination rate (g) of 93–11, *sfp10*, Com, KO, and OE at Stage 12. (h–k) Comparison of the number of grains per main panicle (h), seed setting rate (i), economic yield (j), and biological yield (k) of 93–11, *sfp10*, Com, KO, and OE at the mature stage. Data are presented as the mean ± SD; *n* = 15. Statistical significance was determined using one‐way ANOVA; asterisks indicate significant differences, *p < 0.05; **p < 0.01.

However, significant differences were observed in anther morphology, pollen fertility, and the seed setting rate between the *OsSUT3*‐intact (93–11, OE, and Com) and *OsSUT3*‐deficient lines (*sfp10* and KO) (Figure 2b–e). Compared to *OsSUT3*‐intact lines, *OsSUT3*‐deficient lines exhibited paler and shrunken anthers at Stage 12 of pollen development (Figure 2b). Additionally, pollen fertility and the pollen tube germination rate were significantly lower in *OsSUT3*‐deficient lines than in *OsSUT3*‐intact lines (Figure 2c,d). The pollen fertility of 93–11, OE, Com, *sfp10*, and KO was 95.49%, 97.00%, 95.81%, 64.91%, and 61.74%, respectively (Figure 2f). The pollen tube germination rate of 93–11, OE, Com, *sfp10*, and KO was 52.26%, 54.43%, 52.33%, 11.36%, and 10.01%, respectively (Figure 2g). However, the number of pollen grains did not significantly change among the lines (Figure S2f).

At the maturity stage, both the number of grains per main panicle and seed setting rate were significantly reduced in *OsSUT3*‐deficient lines (*sfp10* and KO) compared to wild‐type 93–11 (WT), with reductions of 23.33% and 25.75% in the number of grains per main panicle and 45.78% and 49.18% in the seed setting rate, respectively. In contrast, these traits did not differ significantly from the WT in the Com and OE lines (Figure 2h,i). In addition, disruption of *OsSUT3* in *sfp10* and KO reduced seed production compared with *OsSUT3*‐intact lines. Both the economic yield (grain yield) and biological yield (total aboveground biomass) of *sfp10* and KO were significantly lower than those of 93–11, OE, and Com (Figure 2j,k). These results indicate that *OsSUT3* positively regulates pollen fertility and the seed setting rate in rice.

### 2.3 *OsSUT3* Is Preferentially Expressed in Late‐Developing Anthers

To confirm the subcellular localization of OsSUT3, OsSUT3‐GFP was transiently expressed in the epidermal cells of *Nicotiana benthamiana* and rice protoplasts, with pm‐mCherry used as a plasma membrane marker. In both systems, OsSUT3‐GFP fluorescence was mainly detected at the cell periphery and showed clear overlap with pm‐mCherry, supporting the plasma membrane localization of OsSUT3 (Figure 3a).

**FIGURE 3:**
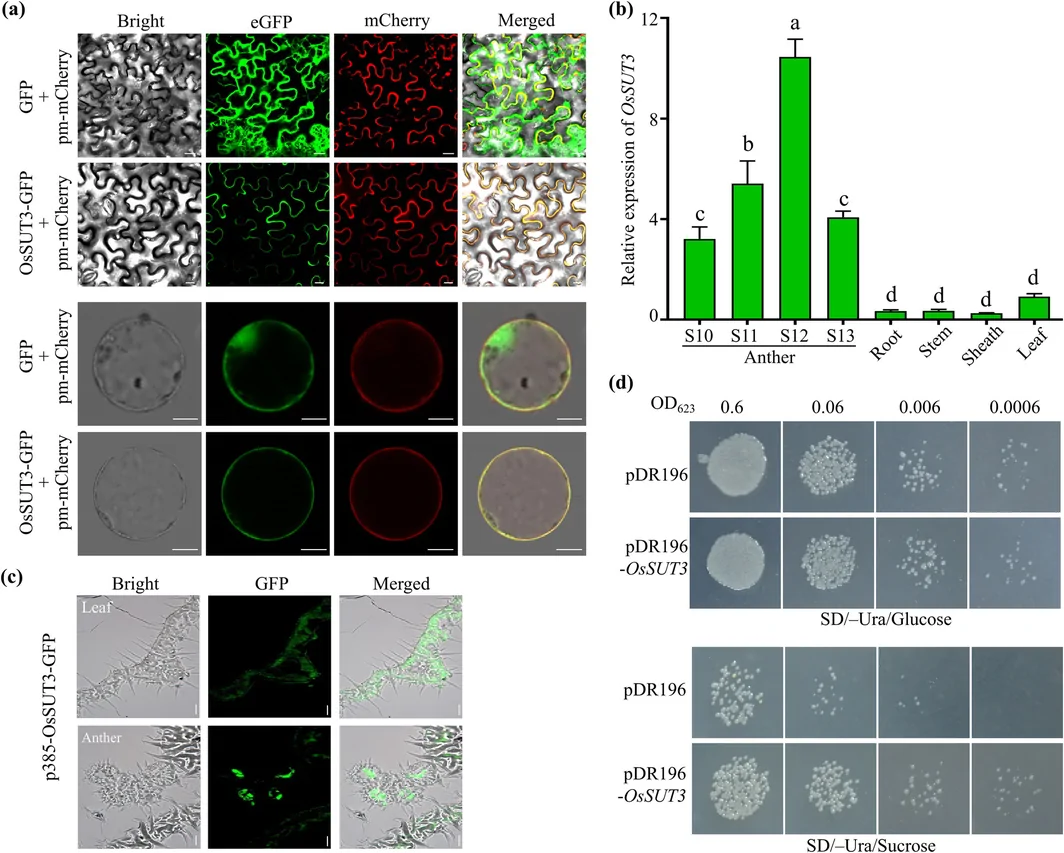
Subcellular localization, spatiotemporal expression pattern, and sucrose transport activity of OsSUT3. (a) Subcellular localization of OsSUT3 in tobacco leaf epidermal cells (bar = 20 μm) and rice protoplasts (bar = 10 μm). (b) RT‐qPCR of the *OsSUT3* gene in 93–11. RNAs were extracted from anthers in later stages of anther development, including Stages 10–13, and from different tissues, including roots, stems, sheaths, and leaves at Stage 12. Data are presented as the mean ± SD; *n* = 3. Statistical significance was determined using one‐way ANOVA; different lowercase letters above bars indicate significant differences among groups (*p* < 0.05). (c) Tissue expression analysis of OsSUT3 in transgenic rice expressing *p385‐OsSUT3‐GFP*. Leaves and anthers at Stage 12 were examined. Bar = 50 μm. (d) Sucrose transport activity analysis of OsSUT3 in *Saccharomyces cerevisiae* strain SUSY7/ura3. The empty vector was transformed as a growth control.

Next, we examined the *OsSUT3* expression pattern during anther development and in different tissues at Stage 12 by reverse transcription quantitative PCR (RT‐qPCR). *OsSUT3* expression was detected from Stage 10; as anther development progressed, its transcript abundance increased, reaching a peak at Stage 12, followed by a decline after pollen maturation (Stage 13) (Figure 3b). In addition, *OsSUT3* was most highly expressed in the anthers, and its expression level was 30.83‐fold higher than in the roots, 30.11‐fold higher than in stems, 41.06‐fold higher than in sheaths, and 11.43‐fold higher than in leaves (Figure 3b), suggesting that *OsSUT3* is preferentially expressed in anthers, especially at Stage 12.

To further verify OsSUT3 potential sites of action in rice, *p385‐OsSUT3‐GFP* was stably expressed in 93–11, and GFP fluorescence was predominantly detected around the vascular bundles in leaves and strongly accumulated in pollen grains at Stage 12 (Figure 3c), consistent with the preferential expression of *OsSUT3* in anthers (Figure 3b).

The molecular function of *OsSUT3* was verified by heterologous expression in a sucrose uptake‐deficient yeast mutant, SUSY7/ura3. The drop test revealed that pDR196‐*OsSUT3* transformants rescued mutant yeast growth on sucrose medium (Figure 3d), indicating that the *OsSUT3* gene regulates sucrose uptake into cells and supplies the energy required for normal growth.

### 2.4 *OsSUT3* Deficiency Impairs Starch Accumulation in Pollen

To determine the function of *OsSUT3* in pollen development, we generated *OsSUT3* KO, OE, and Com transgenic rice lines and assessed *OsSUT3* expression at both the transcript and protein levels (Figure S3). Pollen fertility and cellular‐level morphological changes were analysed in all *OsSUT3*‐intact (WT, OE, and Com) and *OsSUT3*‐deficient (*sfp10* and KO) lines at Stages 10–12 using I_2_‐KI staining under an optical microscope (Figure 4a). No significant differences were observed in the morphological characteristics of anthers and pollen among the lines at Stages 10 (microspore vacuolation) and 11 (first mitotic division of microspores). At these stages, all pollen grains were not stained blue‐purple, suggesting that starch accumulation had not yet begun (Figure 4a). In contrast, at Stage 12, when pollen grains reached maturity, *OsSUT3*‐deficient lines (*sfp10* and KO) showed a significantly higher proportion of pollen grains that weakly stained or unstained pollen grains than *OsSUT3*‐intact (93–11, OE, and Com) lines, suggesting that starch accumulation in pollen and pollen viability significantly decreased due to the loss of *OsSUT3* function (Figure 4a). This reduced viability was supported by the in vivo germination assay (Figure 2d).

**FIGURE 4:**
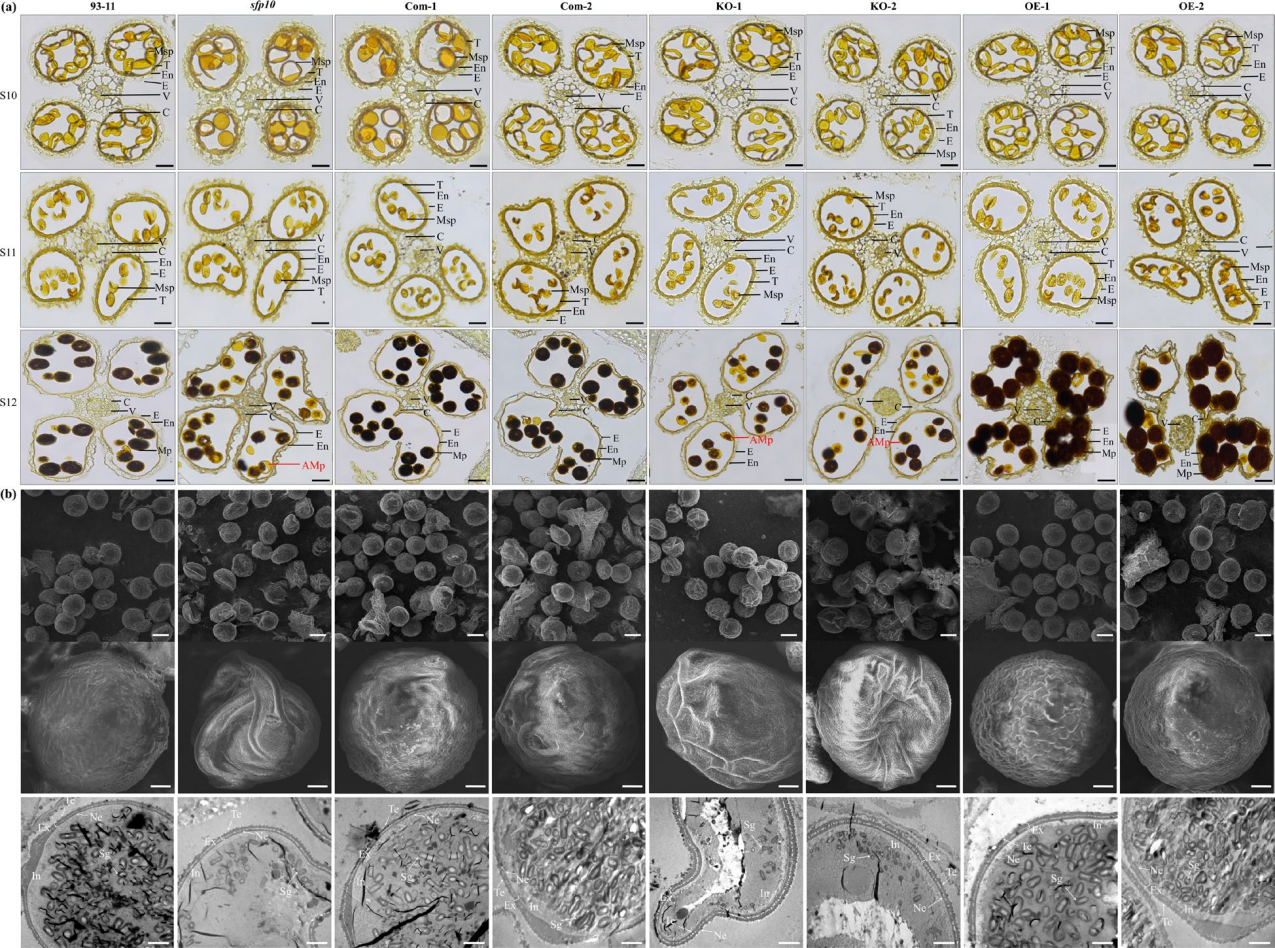
Pollen fertility and pollen morphology of 93–11, *sfp10*, *OsSUT3* complementation (Com), *OsSUT3* knockout (KO), and *OsSUT3* overexpression (OE) lines. (a) Pollen fertility after I_2_‐KI staining of paraffin sections of anthers at Stages 10–12. V, vascular bundle; C, connective tissue; T, tapetum; E, epidermis; En, endothecium; Msp, microspores; Mp, mature pollen; AMp, abnormal mature pollen. Bar = 40 μm. (b) SEM and TEM of pollen grains at Stage 12. Ch, carbohydrates; Ex, exine; In, intine; Ne, nexine; Sg, starch grain; Te, tectum. From top to the bottom, the scale bars indicate 20, 4, and 0.2 μm, respectively.

To determine whether the developmental defects in *OsSUT3*‐deficient pollen were caused by insufficient starch accumulation, we performed ultrastructural analyses on pollen grains from 93 to 11, *sfp10*, Com, KO, and OE lines from Stages 10 to 12 using scanning electron microscopy (SEM) and transmission electron microscopy (TEM). There were no obvious differences in the anther inner surface and microspores among various lines before Stage 12 (Figure S4). At Stage 12, pollen maturation is nearly complete, and the spherical microspores are full of starch and lipid materials (Zhang et al. 2011). At this stage, pollen grains in *OsSUT3*‐intact lines were spherical and fully packed with inclusions, primarily starch granules, and showed signs of maturation (Figure 4b). In contrast, *OsSUT3*‐deficient pollen showed severely impaired accumulation of storage materials, resulting in deflated pollen grains in the anther locule (Figure 4b). These results indicate that *OsSUT3* is required for storage material accumulation during late pollen maturation, particularly starch deposition, which is essential for pollen fertility.

### 2.5 *OsSUT3* Promotes Sucrose Allocation and Carbon Delivery to Anthers During Late Pollen Development

Sucrose is transported from the photosynthetic source organs to the non‐photosynthetic sink anthers, which carry pollen, where it is hydrolyzed by invertase into glucose and fructose to support pollen development and starch biosynthesis (Lee et al. 2022). To reveal the contribution of *OsSUT3* to carbohydrate partitioning during late anther development, we measured the contents of glucose, fructose, sucrose, and starch in the source leaves, sink leaves, stems, and anthers of all *OsSUT3*‐intact (WT, OE, and Com) and *OsSUT3*‐deficient (*sfp10* and KO) lines at Stage 12. In source leaves, there were no significant differences in the glucose, fructose, sucrose, and starch contents among the rice lines (Figure 5a), indicating that the overall carbohydrate status of the photosynthetic source tissue was not altered by *OsSUT3*. In contrast, the sucrose content was increased by 143.72% in sink leaves and by 27.35% in the stems of *OsSUT3*‐deficient lines compared to *OsSUT3*‐intact lines, and the glucose, fructose, and starch contents showed no significant differences among the lines (Figure 5b,c).

**FIGURE 5:**
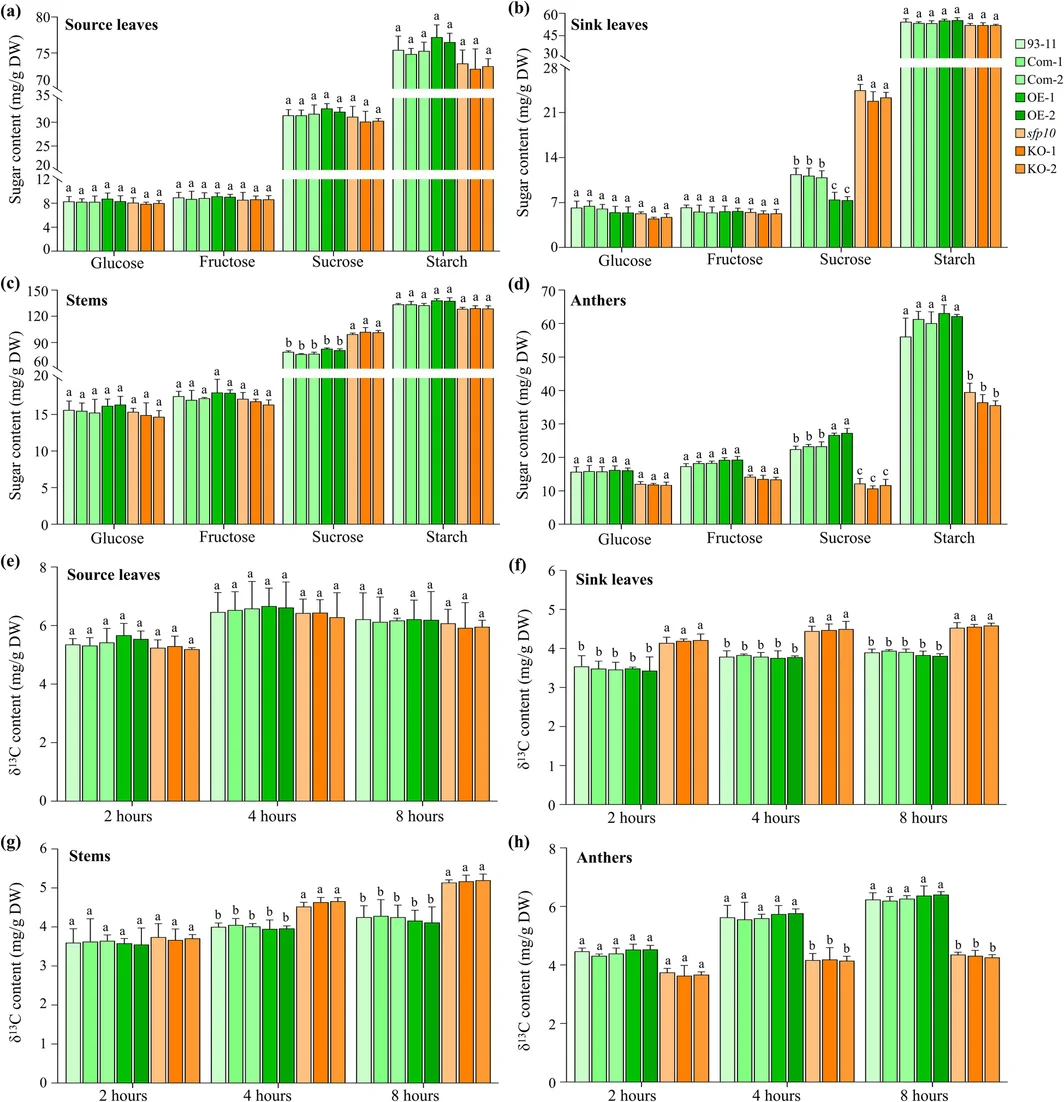
Defects in *OsSUT3* reduce the transport of sucrose and soluble carbon to anthers. (a–d) Glucose, fructose, sucrose, and starch contents in source leaves (a), sink leaves (b), stems (c), and anthers (d) of 93–11, *sfp10*, *OsSUT3* complementation (Com), *OsSUT3* knockout (KO), and *OsSUT3* overexpression (OE) lines at Stage 12. (e–h) Effects of OsSUT3 on soluble carbon fluxes (^13^C tracer contents) in the source leaves (e), sink leaves (f), stems (g), and anthers (h) of 93–11, *sfp10*, Com, KO, and OE at 2, 4, and 8 h after labelling in Stage 12. Data are presented as the mean ± SD; *n* = 3. Statistical significance was analysed by one‐way ANOVA; different lowercase letters above the bars represent significant differences among groups (*p* < 0.05).

In anthers, the soluble sugar and starch contents were significantly lower in the *OsSUT3*‐deficient lines than in the *OsSUT3*‐intact lines. Specifically, the contents of glucose, fructose, sucrose, and starch were reduced by 24.42%, 25.88%, 53.10%, and 38.61%, respectively, in the *OsSUT3*‐deficient lines compared to the *OsSUT3*‐intact lines (Figure 5d). These results suggest that the loss of *OsSUT3* disrupts carbohydrate distribution, leading to insufficient sugar supply and reduced starch accumulation in anthers.

Because metabolite measurements only provide a static view of sugar accumulation, we next performed a ^13^CO_2_ pulse labeling experiment to trace assimilated carbon transport and allocation. As the primary sites of photosynthesis, the source leaves of five rice lines showed minimal differences in ^13^C isotope levels at 2, 4, and 8 h after labeling (Figure 5e), suggesting that carbon fixation in photosynthetic tissues was not significantly affected by *OsSUT3*. In contrast, the ^13^C content in the sink leaves of *OsSUT3*‐deficient lines was significantly greater than that in *OsSUT3*‐intact lines, with increases of 20.21%, 18.03%, and 17.59% at 2, 4, and 8 h, respectively (Figure 5f). In stems, the difference in ^13^C content among lines was not significant at 2 h, but it was significantly greater in *OsSUT3*‐deficient lines than in *OsSUT3*‐intact lines at 4 and 8 h, with significant increases of 15.28% and 22.75%, respectively (Figure 5g). These results indicate that loss of function of *OsSUT3* likely impedes soluble carbon transport, leading to its accumulation in the stems. In anthers, the ^13^C content was significantly lower in *OsSUT3*‐deficient lines than in *OsSUT3*‐intact lines throughout the labeling period, with reductions of 17.19%, 26.03%, and 31.52% at 2, 4, and 8 h, respectively (Figure 5h). Together with the sugar measurements, these results indicate that *OsSUT3* is required for carbon allocation to anthers. The loss of *OsSUT3* resulted in carbon accumulation in sink leaves and stems but reduced carbon delivery to anthers, restricting starch biosynthesis during pollen maturation.

### 2.6 OsWRKY15 Acts as a Transcriptional Activator of *OsSUT3* , Improving Pollen Fertility

To investigate the regulatory mechanism of *OsSUT3*, we performed RNA‐seq on anthers of 93–11 and *sfp10* at Stage 12. The good repeatability of the data was determined by Pearson correlation analysis (93–11: *r* = 0.93 ± 0.06; *sfp10*: *r* = 0.98 ± 0.01; Figure S5). A total of 2459 differentially expressed genes (DEGs; |Fold change| ≥ 2; false discovery rate (FDR) < 0.01) were identified, of which 588, including 7 TFs, were upregulated and 1871, including 84 TFs, were downregulated in *sfp10* (Figure 6a; Tables S2 and S3), suggesting that reduced *OsSUT3* expression is associated with broad transcriptional reprogramming during late pollen development, while its presence may be associated with the coordinated expression of multiple genes that support plant growth and development.

**FIGURE 6:**
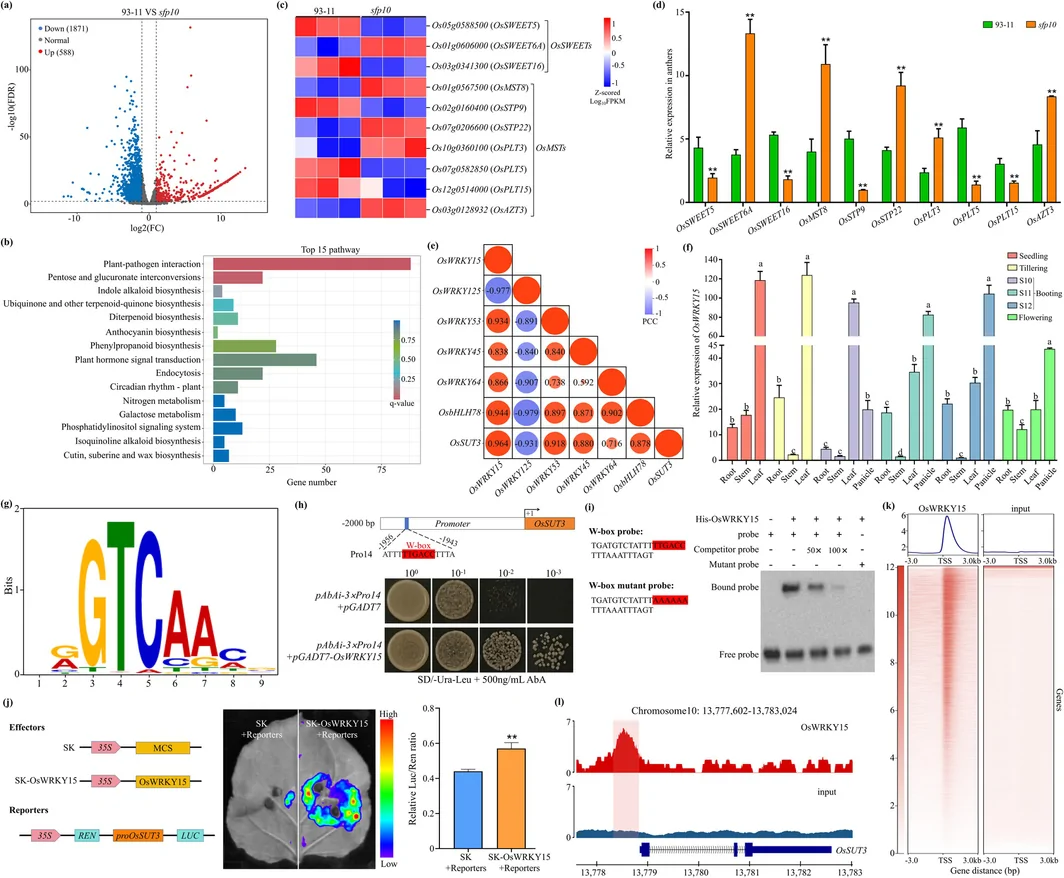
OsWRKY15 positively regulates *OsSUT3* expression. (a) Volcano plot of differentially expressed genes (DEGs) between wild‐type 93–11 and the *sfp10* variant. (b) KEGG enrichment of DEGs. KEGG terms are ranked by the number of enriched genes, with coloured terms indicating associations with pollen development. (c) Expression heat map of 10 sugar transporter DEGs identified by RNA‐seq. The red to blue scale indicates a high to low level of transcript abundance. (d) RT‐qPCR validation of the above 10 sugar transporter genes in Stage 12 anthers. Data are presented as the mean ± SD; *n* = 3. Statistical significance was determined by a *t*‐test; Asterisks indicate significant differences, **p < 0.01. (e) Expression correlation analysis among *OsSUT3* and the OsWRKY transcription factors screened from the top two KEGG enrichment pathways. (f) RT‐qPCR of the *OsWRKY15* gene in 93–11. RNA was extracted from roots, stems, leaves, and panicles at the seedling, tillering, booting (Stage 10, Stage 11, and Stage 12), and flowering stages. Data are presented as the mean ± SD; *n* = 3. Statistical significance was determined using one‐way ANOVA; different lowercase letters above bars indicate significant differences between groups (*p* < 0.05). (g) Prediction of OsWRKY15‐binding site (GGTCAA) in the *OsSUT3* promoter using the PlantPAN website (http://plantpan.itps.ncku.edu.tw). (h) Yeast one‐hybrid assay confirming the interaction between OsWRKY15 and the *OsSUT3* promoter. Tandem repeats of three copies of the Pro14 fragment (containing W‐Box motifs from the *OsSUT3* promoter) were constructed in the pAbAi vector. The *OsWRKY15* coding sequence was constructed in the pGADT7 vector. Transformed yeast cells were grown on SD/‐Ura/−Leu medium with 500 ng/mL AbA. (i) EMSA showing that OsWRKY15 binds to the W‐box motif in the *OsSUT3* promoter. The His‐OsWRKY15 fusion protein and the biotin‐labelled probe containing the W‐box (TTGACC) were used. The unlabeled probe was used as a cold competitor. 50× and 100× represent 50‐fold and 100‐fold molar excess of unlabeled probe, respectively. A mutant probe (AAAAAA) served as the negative control. (j) Dual‐luciferase reporter assay. Effector (SK‐OsWRKY15) and reporter plasmids (proOsSUT3‐LUC) were co‐transformed into *Nicotiana benthamiana* leaves, and the luminescence intensity and luciferase activity were measured. Data are presented as mean ± SD; *n* = 3. Statistical significance was determined using a *t*‐test; asterisks indicate significant differences, **p < 0.01. (k) Heatmap and average metagene profiles of OsWRKY15 ChIP‐seq signals within ±3 kb of the TSSs of OsWRKY15‐bound genes. Input DNA served as the control. (l) ChIP‐seq analysis of OsWRKY15 binding at the *OsSUT3* genomic locus. Normalized ChIP‐seq signal tracks show an enrichment peak in the *OsSUT3* promoter region (Chr10:13777602–13 783 024), whereas the input control shows only background signal. The pink shaded region indicates the major OsWRKY15‐enriched interval overlapping the W‐box‐containing promoter region. The complete list of identified peaks is provided in Table S5.

Kyoto Encyclopedia of Genes and Genomes (KEGG) analysis showed significant enrichment of glycometabolism‐related pathways, including “plant–pathogen interaction” and “pentose and glucuronate interconversions”, based on the DEGs in *sfp10* (Figure 6b). Both pathways encompassed multiple genes that regulate pollen development (e.g., *OsCPK9/21/25/26/29*, *OsCNGC5*, *OsPME1*, *OsPLL3*, and *OSIPP3*) (Kanneganti and Gupta 2009; Wang et al. 2011; Khurana et al. 2013; Wei et al. 2014; Zheng, Yan, et al. 2018; Wen et al. 2019; Ranjan et al. 2022; Kim et al. 2024), all of which were downregulated in *sfp10* (Figure S6a).

Ten sugar transporter genes, comprising seven *MST* and three *SWEET* family members, were identified from the DEGs (Figure 6c). Among these, *OsSWEET6A*, *OsMST8*, *OsSTP22*, *OsAZT3*, *OsSWEET5*, and *OsSTP9* were highly expressed in anthers (Figure S7), similar to *OsSUT3*. We performed RT‐qPCR assays of 6 genes in all experimental rice lines to determine whether the expression of these genes was influenced by *OsSUT3* (Figures 6d and S6b). Compared with the *OsSUT3*‐intact lines, the expression of *OsSWEET6A*, *OsMST8*, *OsSTP22*, and *OsAZT3* was significantly upregulated in *sfp10* and KO lines, whereas the expression of *OsSWEET5* and *OsSTP9* was downregulated in the *OsSUT3*‐deficient lines (Figure S6b). These results suggest that loss of function of *OsSUT3* alters the expression of other sugar transporter genes in rice anthers.

In addition, six TFs related to the TOP2 pathways showed the highest enrichment ratios, of which five belonged to the WRKY family (Table S4). Among them, the transcript level of *OsWRKY15* showed the greatest correlation with that of *OsSUT3* (Figure 6e). Consistent with this, spatiotemporal expression analysis revealed that *OsWRKY15* was preferentially expressed in leaves during vegetative growth but shifted to panicles during reproductive development, with expression peaking at Stage 12 (Figure 6f). This pattern matched the temporal and spatial expression profiles of OsSUT3, supporting a regulatory relationship during late anther development. After analysing this promoter sequence, a typical WRKY TF binding site, the W‐box motif (TTGACC), was identified in the *OsSUT3* promoter region (Figure 6g), suggesting potential transcriptional regulation of *OsSUT3* by WRKY TFs. The Y1H assay demonstrated that OsWRKY15 directly binds to W‐box motifs in the *OsSUT3* promoter region (Figure 6h). EMSA further confirmed the specific binding of His‐OsWRKY15 to the W‐box‐containing promoter fragment, as the shifted band was weakened by an excess of the unlabeled competitor probe but abolished by mutation of the core W‐box sequence (Figure 6i). We next performed a DLR assay to assess the transcriptional regulation of OsWRKY15 on *OsSUT3*. Fluorescence imaging and LUC activity assays revealed that OsWRKY15 activated *OsSUT3* expression (Figure 6j). ChIP‐seq analysis revealed strong OsWRKY15 enrichment around the transcription start sites (TSSs) of its target genes and identified a distinct enrichment peak in the *OsSUT3* promoter region in vivo (Figure 6k,l). Together with our in vitro binding assays, these results confirm that OsWRKY15 directly binds to the *OsSUT3* promoter and activates its expression.

To investigate the biological function of *OsWRKY15* in pollen development and further elucidate the genetic relationship between *OsWRKY15* and *OsSUT3*, we generated CRISPR/Cas9‐mediated *wrky15* lines and then overexpressed or knocked out *OsSUT3* in the *wrky15* background to obtain *wrky15/OsSUT3‐OE* and *wrky15/OsSUT3‐KO* lines, respectively (Figure 7a–d). RT‐qPCR showed that *OsWRKY15* expression was reduced in *wrky15* lines and remained low in the *wrky15/OsSUT3‐OE* and *wrky15/OsSUT3‐KO* lines. Because the CRISPR/Cas9 target sites of *OsWRKY15* were located in the first exon while the RT‐qPCR primers were designed in downstream exons, these RT‐qPCR data were used to assess transcript abundance rather than as direct evidence of gene knockout; successful editing was confirmed by target‐site sequencing (Figure S8). In contrast, *OsSUT3* expression was significantly increased in *wrky15/OsSUT3‐OE* lines but reduced in *wrky15/OsSUT3‐KO* lines (Figure 7e,f). Compared with WT 93–11, *wrky15* lines showed reduced anther starch content, pollen fertility, and seed setting rate, whereas the total pollen number was not significantly altered (Figure 7g–j). Notably, overexpression of *OsSUT3* in the *wrky15* background significantly restored anther starch accumulation, pollen fertility, and seed setting, while knockout of *OsSUT3* further exacerbated these defects (Figure 7g,i,j). These results provide genetic evidence that *OsSUT3* functions downstream of *OsWRKY15* to promote anther starch accumulation, thereby influencing pollen fertility and seed setting in rice.

**FIGURE 7:**
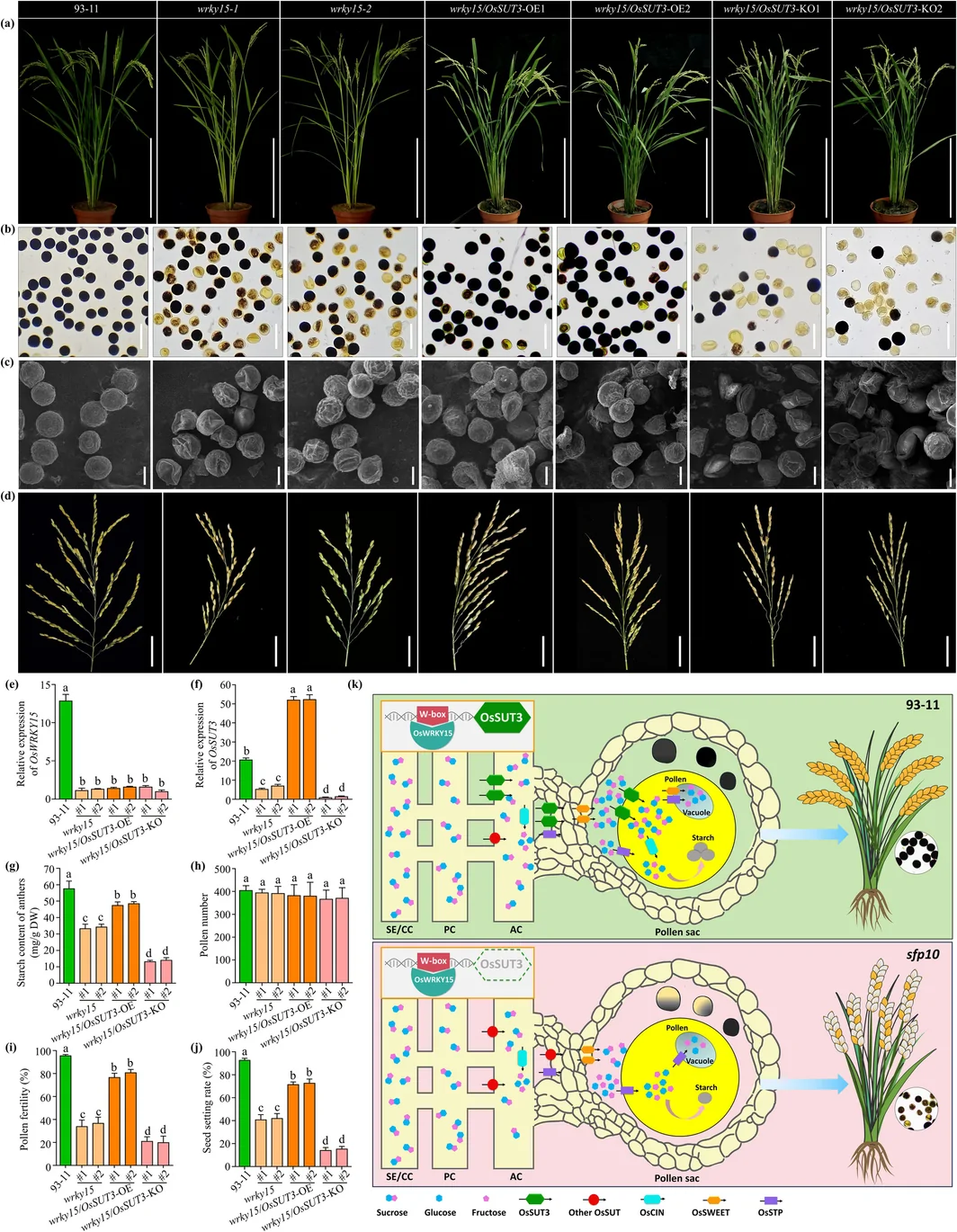
OsSUT3 acts downstream of OsWRKY15 to promote pollen fertility and the seed setting rate in rice. (a) Phenotypes of whole rice plants of 93–11, *OsWRKY15* knockout lines (*wrky15‐1* and *wrky15‐2*), *OsSUT3*‐overexpression lines in the *OsWRKY15* knockout background (*wrky15/OsSUT3‐OE1* and *wrky15/OsSUT3‐OE2*), and *OsSUT3*‐knockout lines in the *OsWRKY15* knockout background (*wrky15/OsSUT3‐KO1* and *wrky15/OsSUT3‐KO2*). Bar = 0.5 m. (b) I_2_‐KI staining of pollen grains of 93–11, *wrky15* lines, *wrky15/OsSUT3‐OE* lines, and *wrky15/OsSUT3‐KO* lines at Stage 12. Bar = 100 μm. (c) SEM of pollen grains at Stage 12. Bar = 20 μm. (d) Phenotypes of the main culm panicles of 93–11, *wrky15* lines, *wrky15/OsSUT3‐OE* lines, and *wrky15/OsSUT3‐KO* lines at the mature stage. Bar = 5 cm. (e,f) Expression of *OsWRKY15* (e) and *OsSUT3* (f) in Stage 12 panicles of 93–11, *wrky15* lines, *wrky15/OsSUT3‐OE* lines, and *wrky15/OsSUT3‐KO* lines. (g–j) Starch content of anthers (g), pollen number (h), pollen fertility (i), and seed setting rate (j) of 93–11, *wrky15* lines, *wrky15/OsSUT3‐OE* lines, and *wrky15/OsSUT3‐KO* lines. Data are presented as the mean ± SD; *n* = 3 for (e–g); *n* = 15 for (h–j). Statistical significance was analysed by one‐way ANOVA; different lowercase letters above the bars represent significant differences between groups (*p* < 0.05). (k) Proposed model by which OsWRKY15 regulates *OsSUT3*, promoting rice pollen fertility and seed setting. *OsSUT3* promotes sucrose unloading into pollen for starch accumulation and increases the seed setting rate.

## 3 Discussion

Rice yield is closely related to pollen fertility (Zhang et al. 2016). Mature and fertile pollen grains accumulate large amounts of storage carbohydrates, particularly starch, which serves as the primary energy source supporting pollen germination and pollen tube elongation (Lee et al. 2016; Chen, Zhang, et al. 2023; Chen, Dong, et al. 2023). The biosynthesis of starch and its accumulation depend on a continuous supply of assimilates, especially sugars (Wu et al. 2023; Tao et al. 2024). To date, except *CsSUT1* in cucumber, none of the SUT mutants of any plant species have been reported to exhibit a starchless pollen phenotype (Sun et al. 2019). Previous studies in rice have revealed the factors contributing to pollen starch accumulation, such as sugar efflux transporters (OsSWEETs) for sucrose export into the apoplastic space of the pollen sac (Wu et al. 2022; Lee et al. 2022), cell wall invertases (OsCINs) for catalysing sucrose hydrolysis in the pollen sac (Oliver et al. 2005; Lee et al. 2024), and hexose transporters (OsSTPs) for hexose import into pollen (Zhang et al. 2010; Deng et al. 2019). *OsSUT3* has previously been shown to be highly expressed in pollen and has been implicated in the transport of sucrose from the apoplast of the pollen sac to rice pollen grains (Hirose et al. 2010). These findings indicate that sucrose is the predominant form of sugar transported to rice pollen, and starch biosynthesis in pollen primarily depends on sucrose as the carbon source. Here, we present evidence supporting the role of *OsSUT3* in pollen starch accumulation during pollen maturation in rice.

In this study, we carried out fine mapping to identify the key determinant of the semi‐sterile pollen and reduced seed setting in the *sfp10* variant and identified *OsSUT3* as the candidate gene responsible for the *sfp10* phenotype (Figure 1). As a plasma membrane‐localized sucrose/H^+^ symporter, OsSUT3 has been reported to play a role in sucrose uptake, which is required for pollen development in rice (Hirose et al. 2010; Lee et al. 2022; Li, Zhang, Wen, et al. 2023). Consistent with this, a significant increase was observed in the number of sterile pollen grains with little or no storage material in the *OsSUT3* knockout lines, but this defect was alleviated in the *OsSUT3* complementation and overexpression lines (Figure 2). Therefore, *sfp10* is a natural variant of the *OsSUT3* gene in rice and may serve as a valuable resource for elucidating the role of *OsSUT3* in pollen development and the mechanism of starch accumulation during the late stages of pollen development.

Starch is the major constituent of the pollen cytoplasm in rice, and its accumulation is crucial for pollen maturation and viability (Wang et al. 2021; Lin et al. 2023). Based on cytological observations, pollen development was normal during vacuolation (Stage 10) and mitosis (Stage 11) stages in *sfp10* (Figures 4a and S4). However, when the pollen reached maturity at Stage 12, subcellular abnormalities became apparent, characterized primarily by collapsed and shrivelled pollen grains (Figure 4b). This phenotype was attributed to the scarcity of starch granules in the cytoplasm caused by the loss of *OsSUT3* function (Figure 4b). Based on sugar profiling and ^13^C tracing (Figure 5), *OsSUT3* plays a central role in coordinating carbon partitioning during late pollen maturation. Insignificant changes in source leaves suggest that *OsSUT3* does not substantially contribute to photosynthetic carbon assimilation. Instead, *OsSUT3* deficiency caused carbon retention in vegetative sink tissues and reduced carbon allocation to anthers, as reflected by the increase in sucrose and ^13^C in sink leaves and stems and decreased soluble sugar, starch, and ^13^C in anthers (Figure 5). Together with its preferential expression in late‐developing anthers (Figure 3b), these findings suggest that *OsSUT3* promotes sucrose import into anther/pollen tissues to sustain starch accumulation, pollen fertility, and final seed setting, similar to the role of the principal translocated sugar in higher plants (Kühn and Grof 2010).

Although monosaccharides can also be imported into pollen via hexose transporters (e.g., STPs and SWEETs) to provide energy and substrates for starch synthesis (Isoda et al. 2022; Koyamatsu et al. 2023; Borghi 2025), the severe pollen defects observed in *OsSUT3*‐deficient plants indicate that these alternative sugar transport pathways cannot fully compensate for impaired sucrose import. Defective *OsSUT3* reduces sucrose uptake in anthers, causing excessive sucrose accumulation in the stems and sink leaves (Figure 5b–d). Besides, ^13^C isotopic tracing revealed that the loss of function of *OsSUT3* also disrupts the transport and distribution of carbon in rice (Figure 5e–h). In maize, the *Cpd1* mutant exhibits excessive starch and soluble sugar accumulation in leaves, primarily due to impaired sucrose export, which disrupts nutrient allocation in the plant, affecting growth and development (Julius et al. 2018). The molecular regulation of *OsSUT3* in carbohydrate partitioning and pollen development requires further investigation in rice.

The expression of most SUT family members has been reported to be regulated by light and hormones (Wu et al. 2021; Sun et al. 2022). Recent studies have further indicated that WRKY TFs are also involved in the regulation of SUT genes. For example, overexpression of *MdWRKY126* in apple enhanced sucrose‐phosphate synthase activity and improved *MdSPS* and *MdSUT* transcription levels (Zhang et al. 2025). Moreover, *OsWRKY71* can alter seed germination time in rice through the repression of *SWEET* and *SUT* gene expression (Bataller et al. 2024). Collectively, these observations suggest that WRKY mediates the regulation of SUTs, but the molecular regulation mechanisms by which WRKY regulates SUT genes are largely unclear. Here, we demonstrated that OsWRKY15 directly interacts with *OsSUT3* and that they positively regulate sucrose uptake, promoting starch accumulation during the later stage of rice pollen development. Based on transcriptome results, gene co‐expression patterns revealed a significant positive correlation between *OsWRKY15* and *OsSUT3* (Figure 6e), and both genes showed reduced expression in *sfp10* anthers compared to 93–11 at Stage 12 of anther development, suggesting that the OsWRKY15 TF is involved in the transcriptional regulation and functional coordination of the *OsSUT3* gene during pollen development. Dynamic spatiotemporal expression analysis revealed that *OsWRKY15* was preferentially expressed in panicles during reproductive development and peaked at Stage 12 in 93–11 (Figure 6f), broadly consistent with the temporal and spatial expression pattern of *OsSUT3*. This coordinated expression supports the regulatory relationship between *OsWRKY15* and *OsSUT3* in late developing anthers.

Mechanistically, Y1H and DLR assays initially indicated that OsWRKY15 bound to the *OsSUT3* promoter and activated its expression (Figure 6h,j). Although the activation effect observed in the DLR assay was modest, it supported the direct interaction between OsWRKY15 and the *OsSUT3* promoter (Figure 6j). This conclusion was reinforced by EMSA and ChIP‐seq results, which provided direct in vitro and in vivo evidence that OsWRKY15 binds the W‐box‐containing region of the *OsSUT3* promoter (Figure 6i,l). Furthermore, transgenic analyses demonstrated that *OsWRKY15* knockout significantly decreased pollen viability and fertility, reducing seed setting (Figure 7i,j). Notably, *OsSUT3* overexpression partially rescued anther starch accumulation, pollen fertility, and seed setting in the *wrky15* background, whereas *OsSUT3* knockout aggravated these defects (Figure 7g,i,j). As the total pollen number was largely unchanged (Figure 7h), the OsWRKY15‐OsSUT3 module appeared to regulate pollen quality and maturation rather than pollen production. Together with evidence from Y1H, EMSA, and ChIP‐seq, these findings support a model in which OsWRKY15 directly activates *OsSUT3* to promote sucrose import into pollen, sustaining starch biosynthesis, pollen fertility, and final seed setting (Figure 7k). However, whether this module is affected by sugar signalling in rice remains to be determined. Disruption of this transport pathway compromises starch accumulation and reduces pollen viability and fertility, confirming that *OsSUT3* is a key target for enhancing pollen fertility and improving the seed setting rate. This finding not only deepens our understanding of carbohydrate partitioning during reproductive development, but it also opens up opportunities for engineering high‐yield, climate‐resilient rice cultivars through the targeted modulation of sucrose transport pathways.

## 4 Materials and Methods

### 4.1 Plant Materials and Growth Conditions

Cultivar 93–11 served as the wild type, and the near‐isogenic line (NIL) *sfp10* was used as the variant for investigations, including comprehensive phenotypic, physiological, cytological, and transcriptomic analyses. BC_4_F_1_ NIL *sfp10* was generated by backcrossing pollen semi‐fertile variant *LSM* (donor parent, *indica* rice) with 93–11 (recurrent parent, *indica* rice) (Li, Zhang, Yang, et al. 2023).

All experimental materials were provided by the Rice Research Institute of Yunnan Agricultural University and cultivated in its experimental greenhouse in Kunming, Yunnan, China (25.13° N, 102.75° E) from March to September with normal field management.

### 4.2 Map‐Based Cloning of *sfp10*

The F_2_ mapping population was constructed by crossing *sfp10* with 93–11. For fine mapping, the population size was expanded to a total of 1285 individuals. We then designed 67 new InDel markers spanning the 398‐kb candidate interval. Based on the IRGSP‐1.0 rice reference genome (https://rapdb.dna.affrc.go.jp), five candidate genes were identified within the refined region, for which 25 cloning primers were designed. All primers were designed using Premier 5.0 and are listed in Table S1.

### 4.3 Vector Construction and Plant Transformation

The 2000‐bp promoter and 1521‐bp coding sequence (CDS) of *OsSUT3* were amplified from 93 to 11 using primer pairs. These fragments were assembled and inserted into binary vector pBWA(V)HII (Biorun, Wuhan, China) to generate _*Pro*_ *OsSUT3‐CDS‐Tnos*. The CDS of *OsSUT3* was fused in‐frame with GFP and inserted into binary vector pBWA(V)HS (Biorun) under the CaMV 35S promoter, generating *35S‐OsSUT3‐GFP*. The 825‐bp CDS of *OsWRKY15* was amplified from 93 to 11 cDNA and fused in‐frame with GFP in the same pBWA(V)HS vector under the 35S promoter, generating *35S‐OsWRKY15‐GFP*. To verify tissue‐specific expression, the 385‐bp core pollen‐specific promoter fragment (p385), identified and characterized in our previous study (Li et al. 2020), was amplified from 93 to 11 genomic DNA and cloned upstream of the *OsSUT3‐GFP* fusion cassette in the same pBWA(V)HS vector, generating the pollen‐specific construct *p385‐OsSUT3‐GFP*.

Knockout vectors for *OsSUT3* and *OsWRKY15* were constructed using the CRISPR/Cas9 system according to Ma et al. (2016). Target sites were designed with the online tool CRISPR‐P 2.0 (http://skl.scau.edu.cn/). The pYLsgRNA‐OsU6a/b vector was digested with the restriction enzyme BsaI to generate sticky ends, to which the annealed oligonucleotides corresponding to the target sequences were ligated to assemble the sgRNA expression cassettes. The cassettes were individually assembled into the pYLCRISPR/Cas9Pubi‐H vector using Golden Gate cloning. The resulting recombinant vectors were designated as *Cas9‐OsSUT3‐H* and *Cas9‐OsWRKY15‐H*.

The *35S‐OsSUT3‐GFP* vector was introduced into 93–11 to generate *OsSUT3*‐overexpressing lines (OE‐1/2). The _*Pro*_ *OsSUT3‐CDS‐Tnos* vector was introduced into *sfp10* to generate complementation lines (Com‐1/2). The *p385‐OsSUT3‐GFP* vector was introduced into 93–11 to generate transgenic lines for tissue‐specific expression analysis. The *35S‐OsWRKY15‐GFP* vector was introduced into 93–11 to generate *OsWRKY15*‐overexpressing lines (*OsWRKY15*‐OE) for ChIP‐seq. The *Cas9‐OsSUT3‐H* and *Cas9‐OsWRKY15‐H* vectors were introduced into 93–11, generating the *OsSUT3* knockout (KO‐1/2) and *OsWRKY15* knockout lines (*wrky15‐1/2*), respectively. To construct genetic epistasis lines, the *Cas9‐OsSUT3‐H* and *35S‐OsSUT3‐GFP* vectors were re‐transformed into the *wrky15‐1* mutant background, generating *wrky15/OsSUT3*‐KO1/2 and *wrky15/OsSUT3*‐OE1/2 lines, respectively. All of the primers used for vector construction are listed in Table S1. All recombinant vectors were delivered into rice via *Agrobacterium tumefaciens* ‐mediated transformation (Qu et al. 2005).

### 4.4 Agronomic Trait Measurement

After physiological maturity of rice plants, key agronomic traits, namely plant height, number of tillers per plant, flag leaf length and width, 1000‐grain weight, number of grains on the main culm panicle, seed setting rate, biological yield (aboveground dry matter weight), and economic yield (grain yield), were measured. The seed setting rate was calculated as follows: (number of filled grains/total number of spikelets per panicle) × 100%. For each tested line, 15 representative plants were randomly evaluated as biological replicates.

### 4.5 Pollen Fertility Determination

The staining of mature pollen grains was performed following the method described by Tao et al. (2019). Briefly, pollen grains were shed onto a glass slide, stained with 1% (w/v) I_2_‐KI solution for 5 min, and then observed and photographed under an optical microscope (Nikon SMZ1000, Tokyo, Japan).

### 4.6 Aniline Blue Staining of Pollen Germination in Vivo

In vivo aniline blue staining of pollen tubes was performed following the method described by Yu et al. (2019), with minor adjustments. Pistils were collected 30 min post‐anthesis or manual pollination, fixed in Carnoy's solution at 25°C for 2 h, rinsed with ddH_2_O five times, softened in 1 M NaOH at 55°C for 30 min, and stained overnight with aniline blue in the dark. Fluorescence imaging was performed using a fluorescence microscope (Carl Zeiss Axiostar Plus, Oberkochen, Germany).

### 4.7 Morphological and Cytological Observations

The morphology of whole plants and panicles was recorded using a digital camera (Nikon D5600, Tokyo, Japan). Fresh spikelets, florets, and anthers were visualized under a stereomicroscope (Olympus SZ2‐ILST, Tokyo, Japan). Anther developmental stages were determined according to the cytological criteria described by Zhang et al. (2011). Specifically, Stage 10 was characterized by enlarged, highly vacuolated round microspores with an intact tapetum; Stage 11 was defined by the first mitotic division of microspores, reduced vacuolation, and progressive tapetal degeneration; Stage 12 featured mature pollen with complete starch accumulation and the absence of the tapetum. Fifteen biological replicates were observed for each morphological assessment.

For paraffin sectioning, anther transverse sections were prepared as described by Chen, Zhang, et al. (2023). Briefly, I_2_‐KI stained anthers were fixed, dehydrated, embedded in paraffin, and sectioned at 5–6 μm using a rotary microtome (Leica RM2235, Wetzlar, Germany). Sections were subsequently imaged under a fluorescence microscope in bright‐field mode (Carl Zeiss Axio Observer.A1, Oberkochen, Germany). The observations were based on fifteen anthers from independent plants.

SEM and TEM were performed as described by Liu et al. (2017). For SEM, samples were fixed in Formalin‐Aceto‐Alcohol (FAA) solution, dehydrated through a graded ethanol series, critical‐point dried (Leica EM CPD300, Wetzlar, Germany), and examined with a scanning electron microscope (Hitachi FlexSEM‐1000, Tokyo, Japan). For TEM, samples were fixed in a mixture of 3.5% glutaraldehyde (w/v) and 2% paraformaldehyde (w/v) in 0.1 M PBS (pH 7.2), post‐fixed with 2% OsO_4_ (w/v), dehydrated, and embedded in Spurr's resin (Electron Microscopy Sciences, Hatfield, PA, USA). Ultrathin sections (50–70 nm) were prepared using an ultramicrotome (Leica EM UC7, Wetzlar, Germany) and observed under a transmission electron microscope (JEOL JEM‐1200EXII, Tokyo, Japan). Fifteen independent anther samples were observed for each technique.

### 4.8 Total RNA Extraction and RT‐qPCR Analysis

Total RNA was extracted using TRIzol reagent (Tiangen DP424, Beijing, China). RNA integrity was verified using 1.2% denaturing agarose gel electrophoresis (XHLY 111860, Beijing, China), and RNA concentration and purity were determined spectrophotometrically using a NanoDrop 2000 spectrophotometer (Thermo Fisher Scientific, MA, USA).

Total RNA was reverse‐transcribed using the FastKing‐RT Kit (Tiangen KR118, Beijing, China). RT‐qPCR was performed in 20‐μL reactions containing SYBR Green SuperReal Premix Plus (Tiangen FP205, Beijing, China) on a Real‐Time PCR System (Bio‐Rad CFX96, CA, USA). The thermal cycling conditions were 40 cycles of 95°C for 15 s and 60°C for 30 s. RT‐qPCR primers designed with Premier 5.0 (Table S1) were used, with *β‐actin* serving as the endogenous control. The relative gene expression levels were calculated using the 2^−∆∆Ct^ method (Arocho et al. 2006) with three biological replicates.

### 4.9 Western Blot

Total proteins in rice anthers were extracted using a plant protein extraction kit (Solarbio BC3720, Beijing, China). The protein concentrations were determined using a Bradford Protein Assay Kit (Solarbio PC0010, Beijing, China). Equal protein aliquots (20–30 μg) were separated by 12.5% (w/v) sodium dodecyl sulfate‐polyacrylamide gel electrophoresis (SDS‐PAGE) using a pre‐cast gel system (Tsingke TSW0201‐04, Beijing, China) and transferred to polyvinylidene fluoride (PVDF) membranes (Solarbio IPVH00010, Beijing, China) via semi‐dry electroblotting.

Membranes were incubated at 4°C overnight with the following primary antibodies: custom rabbit polyclonal anti‐OsSUT3 antibody (1:500; ABclonal, Wuhan, China) generated against synthetic peptides DAARAPAEEGGVRASA‐C and GGDGKGKAPPQIS‐C derived from unique OsSUT3 epitopes; and anti‐β‐actin (1:7000; ABclonal AC009, Wuhan, China). Membranes were then probed with HRP‐conjugated anti‐rabbit IgG secondary antibody (1:8000; ABclonal AS023, Wuhan, China) at room temperature for 1 h. Protein signals were detected using SuperSignal West Pico chemiluminescent substrate (Thermo 34 577, MA, USA) and visualized using a ChemiDoc MP Imaging System (Bio‐Rad 12 003 154, CA, USA). Three biological replicates were analysed.

### 4.10 Subcellular Localization Analysis

The full‐length CDS of *OsSUT3* was fused in‐frame upstream of GFP in the pBWA(V)HS vector under the CaMV 35S promoter, generating the *35S‐OsSUT3‐GFP* construct. Plasma membrane marker pm‐mCherry (pBI121‐mCherry‐PM; Coolaber, Beijing, China) was used to visualize the cell periphery. The recombinant plasmids (OsSUT3‐GFP and pm‐mCherry) were co‐transformed into *Agrobacterium tumefaciens* strain GV3101 and co‐infiltrated into the leaves of 4‐week‐old *Nicotiana benthamiana* (Yang et al. 2014). As a negative control, empty vector 35S‐GFP (expressing free GFP) was co‐infiltrated with pm‐mCherry following the same procedure. After incubation in the dark for 48 h, fluorescence signals were observed using a confocal laser scanning microscope (Zeiss LSM 880, Oberkochen, Germany). GFP and mCherry were excited at 488 and 561 nm, respectively.

For transient expression in rice protoplasts, the same *35S‐OsSUT3‐GFP* construct was used. Protoplasts were isolated from the leaf sheaths of 12‐d‐old rice seedlings and transformed with plasmids via PEG‐mediated transfection, as described by Zhang et al. (2023). The plasma membrane marker pm‐mCherry was co‐transformed as a control. After incubation, fluorescence signals were observed using a confocal laser scanning microscope (Zeiss LSM 880).

We used transgenic lines expressing the *p385‐OsSUT3‐GFP* construct to examine the tissue‐specific accumulation and subcellular localization of OsSUT3 in rice. Leaves and anthers were collected separately at developmental Stage 12. Samples were fixed in 4% paraformaldehyde overnight at 4°C, dehydrated through an ethanol series, embedded in paraffin, and sectioned into 8‐μm slices using a microtome (Leica RM2235, Wetzlar, Germany). Sections were mounted on slides and observed directly under a confocal laser scanning microscope (Zeiss LSM 880) without additional staining. GFP fluorescence was excited at 488 nm and detected at 500–530 nm.

### 4.11 Heterologous Expression of *OsSUT3* in Yeast

The cDNA of *OsSUT3* was cloned into the yeast expression vector pDR196 and transformed into the sucrose uptake mutant *Saccharomyces cerevisiae* SUSY7/ura3. Functional complementation was assessed using a drop assay as previously described (Ma et al. 2019). Yeast carrying the pDR196 empty vector served as the control. Transformants were grown in liquid synthetic deficient (SD)/−uracil medium with 2% glucose. Serial dilutions of the cell suspensions were spotted onto solid SD/−uracil medium containing either 2% sucrose or 2% glucose (control). Growth was photographed after incubation at 30°C for 2–4 days.

### 4.12 Determination of Sucrose, Glucose, Fructose, and Starch Contents

For carbohydrate analysis, source and sink leaves were collected from plants with anther developmental Stages 11 and 12. Source leaves were defined as photosynthetically active flag leaves. Sink leaves were defined as elongating flag leaves wrapped in leaf sheaths, as previously described (Aoki et al. 2003). The contents of sucrose, glucose, and fructose in the source leaves, sink leaves, stems, and anthers at anther developmental Stages 11 and 12 were determined using high‐performance liquid chromatography (HPLC) (Chen et al. 2019). The HPLC system consisted of a Waters 2695 chromatograph (Waters, MA, USA) coupled to a Waters 2424 evaporative light scattering detector. Separation was achieved on an amino column (Dikma Platisil‐NH2, Beijing, China) using an acetonitrile/water mixture (70/30, v/v) as the mobile phase at 1.0 mL/min. The column temperature was maintained at 35°C, and the injection volume was 10 μL.

The starch content in the same set of tissues was determined colorimetrically using the anthrone‐sulfuric acid method (Hansen and Moller 1975). Dried samples (0.1 g) were hydrolyzed with 80% ethanol and reacted with 2% anthrone reagent in concentrated H_2_SO_4_. The absorbance was measured using a spectrophotometer (Shimadzu UV‐2600, Kyoto, Japan) at 620 nm.

### 4.13 ^13^C Pulse Labeling

Source and sink leaves were defined as described in the carbohydrate analysis. The ^13^C pulse labeling experiment followed established methods (Bai et al. 2021). Briefly, at 10:00 AM, rice plants at Stage 12 were enclosed in an airtight glass chamber (0.8 × 0.8 × 2.0 m). A ^13^CO_2_ atmosphere was generated within the chamber by injecting 50 mL of 1 M HCl into 2 g of NaH^13^CO_3_ (99 atom% ^13^C). Tissue samples (source leaves, sink leaves, stems, and anthers) were collected before labeling (0 h control) and at 2, 4, and 8 h after labeling. Harvested tissues were flash‐frozen in liquid nitrogen, oven‐dried at 80°C for 48 h, and homogenized into a fine powder using a cryogenic mill. The δ^13^C values were determined by isotope ratio mass spectrometry (Thermo MAT 253, MA, USA) using the international Pee Dee Belemnite (PDB) standard.

δ^13^C was calculated as follows:

$$\delta^{13}C\;\left(‰\right)=\left(\frac{R_{\mathrm{sample}}}{R_{\mathrm{standard}}}-1\right)\times 1000$$

where $R_{\text{sample}}$ is the ^13^C/^12^C ratio in the samples and $R_{\text{standard}}$ (0.011237) is the isotopic ratio of ^13^C/^12^C in Pee Dee Belemnite (PDB).

^13^C atom% excess was calculated as follows:

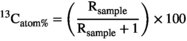




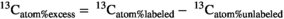

where ^13^C_atom% excess_ represents the percentage of ^13^C atoms among the total carbon atoms present in the labelled and unlabeled samples.

The ^13^C content was estimated as follows:

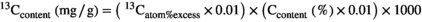




### 4.14 RNA‐Seq Analysis

Stage 12 anthers from 93 to 11 and *sfp10* with three biological replicates for each genotype were submitted to Biomarker Technology Co. Ltd. (Beijing, China) for RNA‐seq. All subsequent bioinformatics analyses, including differential expression, GO enrichment, and KEGG pathway analyses, were performed on the BMKCloud platform (www.biocloud.net). To identify candidate regulators, Pearson correlation analysis was conducted between the FPKM values of *OsSUT3* and those of the significantly enriched TFs (Table S4). Pearson correlation coefficients and two‐tailed *p*‐values were calculated using SPSS Statistics 26. A *p*‐value < 0.05 was considered statistically significant.

### 4.15 Yeast One‐Hybrid (Y1H) Assay

Transcriptional regulation of *OsSUT3* was analysed using the pAbAi/pGADT7 dual‐plasmid system (Coolaber YH1001, Beijing, China). A 14‐bp promoter segment (−1956 to −1943) containing the W‐box motif (5′‐TTGACC‐3′), designated Pro14, was synthesized as three tandem repeats. This trimeric Pro14 fragment was cloned into the multiple cloning site (MCS) of the pAbAi vector (carrying an AbAr resistance marker) to generate the bait plasmid *pAbAi‐3 × Pro14*. Autoactivation testing was performed in the *Saccharomyces cerevisiae* Y1HGold yeast strain, and the Aureobasidin A (AbA) minimal inhibitory concentration was 500 ng/mL.

The 825‐bp *OsWRKY15* CDS was amplified from cDNA of 93 to 11 using PCR with gene‐specific primers (Table S1) and cloned into the pGADT7 vector, generating the prey plasmid pGADT7‐OsWRKY15. The bait and prey plasmids were co‐transformed into Y1HGold yeast competent cells (Coolaber CC308, Beijing, China) using the LiAc/SS carrier DNA/PEG method (Gietz and Schiestl 2007). Transformants were selected on SD/‐Leu/‐Ura agar medium (Coolaber PM2272, Beijing, China) supplemented with 500 ng/mL AbA after incubation at 30°C for 3 days. Positive colonies exhibiting AbA resistance were validated by colony PCR and sequencing (Sangon Biotech, Shanghai, China). Negative controls consisting of empty vectors (pAbAi‐3 × Pro14 + pGADT7) were included, and there were three biological replicates for each experiment.

### 4.16 Electrophoretic Mobility Shift Assay (EMSA)

The CDS of *OsWRKY15* was cloned into the pET‐32a vector and expressed in *Escherichia coli* BL21 (DE3). Recombinant His‐OsWRKY15 protein was purified using Ni‐NTA agarose (Thermo, MA, USA). Biotin‐labelled DNA probes (30 bp) containing the wild‐type W‐box motif or its mutant version were synthesized (Sangon Biotech, Shanghai, China); their sequences are listed in Table S1. Binding reactions were performed using a LightShift Chemiluminescent EMSA Kit (Thermo 20 148, MA, USA). For competition assays, 50‐ or 100‐fold molar excesses of unlabeled probe were added. Reaction mixtures were resolved on 6% native polyacrylamide gels, transferred to nylon membranes, and detected via chemiluminescence using a ChemiDoc MP Imaging System (Bio‐Rad, CA, USA).

### 4.17 Dual‐Luciferase Reporter (DLR) Assay

The 2000‐bp promoter fragment of *OsSUT3* was cloned into the MCS of the pGreenII 0800‐Luc vector (NovoPro V010545, Shanghai, China) to construct reporter plasmid _Pro_OsSUT3‐LUC. The 825‐bp *OsWRKY15* CDS was inserted into the MCS of the *pGreenII‐62SK* vector (NovoPro V012539, Shanghai, China) to generate effector plasmid SK‐OsWRKY15. All primers used are listed in Table S1.

Both plasmids were mixed with the internal control plasmid pRL‐TK (NovoPro V011444, Shanghai, China) at a ratio of 2:2:1 and introduced into *Agrobacterium tumefaciens* GV3101 (Coolaber AT407, Beijing, China) using electroporation. The leaves of 4‐week‐old tobacco plants were infiltrated with the *Agrobacterium* suspension (OD_600_ = 0.6) using a needleless 1‐mL syringe. After 48 h of incubation in the dark, the leaves were sprayed with 1 mM D‐luciferin (Coolaber CL6930, Beijing, China) and the luminescence intensity was visualized using the cryo‐cooled CCD imaging system (Boluteng, PlantView100, Guangzhou, China).

Leaf discs (1 cm diameter) from infiltrated areas were homogenized in passive lysis buffer (Beyotime RG027‐1, Shanghai, China) and centrifuged (12 000 × g, 5 min). The firefly luciferase (LUC) and Renilla luciferase (REN) activities in the supernatant were measured using a DLR Assay Kit (Beyotime RG027, Shanghai, China) on a GloMax Discover luminometer (Promega, WI, USA). The promoter activity was calculated as the Firefly/Renilla ratio. Controls included empty vectors (*SK +* _*Pro*_ *OsSUT3‐LUC*) with three biological replicates.

### 4.18 Chromatin Immunoprecipitation Sequencing (ChIP‐Seq)

ChIP‐seq was performed using Stage 12 anthers from 35S‐OsWRKY15‐GFP transgenic rice following the method described by Kaufmann et al. (2010). Tissues were cross‐linked with 1% formaldehyde for 15 min, quenched with 0.125 M glycine, and ground in liquid nitrogen. Chromatin was extracted and sonicated into 200–500 bp fragments using a Bioruptor Plus (Diagenode, Liège, Belgium). Immunoprecipitation (IP) was carried out overnight at 4°C with 5 μg of anti‐GFP antibody (Abcam ab290, Cambridge, UK). Protein A/G magnetic beads (Invitrogen, CA, USA) were added to capture the immunocomplexes.

After sequential low‐salt, high‐salt, LiCl, and TE washes, cross‐links were reversed at 65°C in the presence of NaCl, and DNA was purified using a MinElute PCR Purification Kit (QIAGEN, Hilden, Germany). A 1% aliquot of sheared chromatin was retained as the input control and processed in parallel.

Libraries were prepared using a NEBNext Ultra II DNA Library Prep Kit (New England Biolabs, MA, USA) and sequenced on the Illumina NovaSeq 6000 platform by Biomarker Technology (Beijing, China), generating approximately 20 million reads per sample across 2 independent biological replicates. Raw reads were trimmed with Trim_Galore (Q20 threshold) and aligned to the *Oryza sativa* reference genome (IRGSP‐1.0) using Bowtie2 (v2.4.2), and uniquely mapped reads (MAPQ > 20) were retained using SAMtools (v1.4.1). Peaks were called using MACS2 (v2.1.7) with the input as the control (parameters: bandwidth = 300, *q*‐value < 0.01; Zhang et al. 2008). Motif analysis was performed with MEME‐ChIP, and enriched peaks were visualized using IGV and deepTools.

### 4.19 Statistical Analysis

Data were compiled using Microsoft Excel 2019 and analysed using IBM SPSS Statistics 26. Figures were generated using GraphPad Prism 8.0 and Adobe Illustrator 2021. Comparisons between two groups were performed using a two‐tailed Student's *t*‐test. For comparisons among three or more groups, one‐way analysis of variance (ANOVA) was conducted, followed by Tukey's honestly significant difference test.

## Author Contributions

D.L. and J.W. designed the research and conceived and supervised the project. Q.L. and D.L. performed data analysis and revised the manuscript. Q.L. and C.Z. performed experiments. S.W., J.C., Y.S., W.M., and Y.Y. assisted with some experiments. Q.Y. and L.C. provided technical assistance and contributed ideas for the manuscript. All authors read and approved the manuscript.

## Funding

This work was supported by the Yunnan Agricultural Basic Research Joint Special Project‐General Program (202301BD070001‐121) and the Major Science and Technology Special Project of Yunnan Province (202402AE090026‐04).

## Conflicts of Interest

The authors declare no conflicts of interest.

## Supporting information


**Figure S1:** Cloning and sequence analysis of candidate genes. (a) Electrophoresis patterns of PCR amplification of 5 candidate genes. (b) Comparison of *Os10g0404500* (*OsSUT3*) gene sequencing peaks between 93 and 11 and *sfp10*. (c) Comparison of OsSUT3 protein sequences between 93 and 11 and *sfp10*.
**Figure S2:** Agronomic trait analysis of 93–11, *sfp10*, complementation (Com), knockout (KO) and overexpression (OE) lines. (a) Schematic representation of the *OsSUT3* CRISPR null alleles. The nucleotides around the CRISPR/Cas9 edited sites are compared between 93 and 11 (WT) and KO. (b–e) Comparison of plant height (b), number of tillers (c), flag leaf length (d) and flag leaf width (e) in 93–11, *sfp10*, Com, KO and OE at the filling stage. (f) Comparison of the number of pollen grains in 93–11, *sfp10*, Com, KO and OE at Stage 12. (g–i) Comparison of the 1000‐grain weight (g), economic yield (h) and biological yield (i) in 93–11, *sfp10*, Com, KO and OE at the mature stage. Data are presented as the mean ± SD; *n* = 15. Statistical significance was determined using one‐way ANOVA.
**Figure S3:** OsSUT3 expression at the transcriptional and protein levels in the anthers of 93–11, *sfp10*, complementation (Com), knockout (KO) and overexpression (OE) lines at Stage 12. (a) Relative expression of *OsSUT3* determined by RT‐qPCR. Data are presented as the mean ± SD; *n* = 3. Statistical significance was determined by one‐way ANOVA; different lowercase letters indicate significant differences between groups (*p* < 0.05). (b) OsSUT3 protein expression analysed by Western blot.
**Figure S4:** Phenotypic analysis of pollen development at Stages 10 and 11 in 93–11, *sfp10*, complementation line (Com), knockout line (KO) and overexpression line (OE). (a–b) Pollen morphology and ultrastructure at Stage 10 (a) and Stage 11 (b). Left panels: Scanning electron microscopy (SEM) showing pollen surface morphology. Right panels: Transmission electron microscopy (TEM) revealing internal ultrastructure. Ex, exine; Cp, cytoplasm. From the left to the right, the scale bar indicated 20, 4 and 0.2 μm.
**Figure S5:** Heatmap diagram of inter‐sample correlation analysis. Note: colour blocks indicate the correlation coefficient values; blue indicates a lower correlation and red represents a higher correlation coefficient between samples.
**Figure S6:** pbi70738‐sup‐0001‐Figures.docx. *OsSUT3* mutation disrupts the expression of pollen development‐related and sugar transport genes. (a) Annotation of genes related to pollen development and their expression in 93–11 and *sfp10*. (b) RT‐qPCR of sugar transport DEGs. Data are presented as the mean ± SD; *n* = 3. Statistical significance was determined using one‐way ANOVA; different lowercase letters indicate significant differences between groups (*p* < 0.05).
**Figure S7:** Expression patterns of *OsSUT3*‐associated sugar transporter genes: (a) *OsSWEET5*, (b) *OsSWEET6A*, (c) *OsMST8*, (d) *OsSTP9*, (e) *OsSTP22*, (f) *OsAZT3*, (g) *OsPLT15*, (h) *OsPLT5*, (i) *OsPLT3* and (j) *OsSWEET16*. The red circle indicates high expression in the panicle at the pollen maturity stage. The expression patterns were retrieved from Bio‐Analytic Resource for Plant Biology (http://bar.utoronto.ca).
**Figure S8:** Schematic representation of CRISPR/Cas9‐edited sites of *OsWRKY15* and *OsSUT3* in single and double knockout lines. (a) Mutation sites of *OsWRKY15* in two independent wrky15 single knockout lines (*wrky15‐1* and *wrky15‐2*). (b) Mutation sites of both *OsWRKY15* (upper panel) and *OsSUT3* (lower panel) in two independent *wrky15/OsSUT3* double knockout lines (*wrky15/OsSUT3*‐KO1 and *wrky15/OsSUT3*‐KO2). Red dashes indicate deleted bases; red letters indicate inserted or substituted bases. The protospacer adjacent motif (PAM) is highlighted in blue.

**Table S1:** Primers used in this study.
**Table S2:** DEGs identified by RNA‐seq comparing 93–11 and *sfp10* anthers.
**Table S3:** TFs identified in DEGs.
**Table S4:** Genes enriched in the “plant–pathogen interaction” and “pentose and glucuronate interconversions” pathways.
**Table S5:** List of OsWRKY15 binding peaks identified by ChIP‐seq in rice anthers.

## Data Availability

The data that supports the findings of this study are available in the Supporting Information of this article. Accession numbers: Gene information in this study can be obtained in the Rice Annotation Project (RAP) database under the following accession numbers: *OsSUT3* ( *Os10g0404500* ); *OsWRKY15* ( *Os01g0656400* ); *OsWRKY125* ( *Os11g0684100* ); *OsWRKY53* ( *Os05g0343400* ); *OsWRKY45* ( *Os05g0322900* ); *OsWRKY64* ( *Os12g0116700* ); *OsSWEET5* ( *Os05g0588500* ); *OsSWEET6A* ( *Os01g0606000* ); *OsAZT3* ( *Os03g0128932* ); *OsMST8* ( *Os01g0567500* ); *OsSTP9* ( *Os02g0160400* ); and *OsSTP22* ( *Os07g0206600* ).
