# Supplementary material for: The OsWRKY15‐OsSUT3 Regulatory Module Enhances Rice Pollen Fertility by Mediating Sucrose Allocation to Anthers
Source: Plant Biotechnol J. 2026 Aug 4;24(11):5684–701. doi: 10.1111/pbi.70738 (PMC13439041; doi:10.1111/pbi.70738)
Supplement: Supplementary file 1 [file PBI-24-5684-s001.docx]

**Figure S1** Cloning and sequence analysis of candidate genes. (a) Electrophoresis patterns of PCR amplification of 5 candidate genes. (b) Comparison of *Os10g0404500* (*OsSUT3*) gene sequencing peaks between 93-11 and *sfp10*. (c) Comparison of OsSUT3 protein sequences between 93-11 and *sfp10*

**Figure S2** Agronomic trait analysis of 93-11, *sfp10*, complementation (Com), knockout (KO), and overexpression (OE) lines. (a) Schematic representation of the *OsSUT3* CRISPR null alleles. The nucleotides around the CRISPR/Cas9 edited sites are compared between 93-11 (WT) and KO. (b–e) Comparison of plant height (b), number of tillers (c), flag leaf length (d), and flag leaf width (e) in 93-11, *sfp10*, Com, KO, and OE at the filling stage. (f) Comparison of the number of pollen grains in 93-11, *sfp10*, Com, KO, and OE at Stage 12. (g-i) Comparison of the 1000-grain weight (g), economic yield (h), and biological yield (i) in 93-11, *sfp10*, Com, KO, and OE at the mature stage. Data are presented as the mean ± SD; n = 15. Statistical significance was determined using one-way ANOVA.

**Figure S3** OsSUT3 expression at the transcriptional and protein levels in the anthers of 93-11, *sfp10*, complementation (Com), knockout (KO), and overexpression (OE) lines at Stage 12. (a) Relative expression of *OsSUT3* determined by RT-qPCR. Data are presented as the mean ± SD; n = 3. Statistical significance was determined by one-way ANOVA; different lowercase letters indicate significant differences between groups (*P* < 0.05). (b) OsSUT3 protein expression analyzed by Western blot.

**Figure S4** Phenotypic analysis of pollen development at Stages 10 and 11 in 93-11, *sfp10*, complementation line (Com), knockout line (KO), and overexpression line (OE). (a-b) Pollen morphology and ultrastructure at Stage 10 (a) and Stage 11 (b). Left panels: Scanning electron microscopy (SEM) showing pollen surface morphology. Right panels: Transmission electron microscopy (TEM) revealing internal ultrastructure. Ex, exine; Cp, cytoplasm. From the left to the right, the scale bar indicated 20, 4, and 0.2 μm.

**Figure S5** Heatmap diagram of inter-sample correlation analysis. Note: color blocks indicate the correlation coefficient values; blue indicates a lower correlation, and red represents a higher correlation coefficient between samples.

**Figure S6** *OsSUT3* mutation disrupts the expression of pollen development-related and sugar transport genes. (a) Annotation of genes related to pollen development and their expression in 93-11 and *sfp10*. (b) RT-qPCR of sugar transport DEGs. Data are presented as the mean ± SD; n = 3. Statistical significance was determined using one-way ANOVA; different lowercase letters indicate significant differences between groups (*P* < 0.05).

**Figure S7** Expression patterns of *OsSUT3*-associated sugar transporter genes: (a) *OsSWEET5*, (b) *OsSWEET6A*, (c) *OsMST8*, (d) *OsSTP9*, (e) *OsSTP22*, (f) *OsAZT3*, (g) *OsPLT15*, (h) *OsPLT5*, (i) *OsPLT3*, and (j) *OsSWEET16*. The red circle indicates high expression in the panicle at the pollen maturity stage. The expression patterns were retrieved from Bio-Analytic Resource for Plant Biology ([http://bar.utoronto.ca](http://bar.utoronto.ca/efprice/cgi-bin/efpWeb.cgi)).

**Figure S8** Schematic representation of CRISPR/Cas9-edited sites of *OsWRKY15* and *OsSUT3* in single and double knockout lines. (a) Mutation sites of *OsWRKY15* in two independent wrky15 single knockout lines (*wrky15-1* and *wrky15-2*). (b) Mutation sites of both *OsWRKY15* (upper panel) and *OsSUT3* (lower panel) in two independent *wrky15/OsSUT3* double knockout lines (*wrky15/OsSUT3*-KO1 and *wrky15/OsSUT3*-KO2). Red dashes indicate deleted bases; red letters indicate inserted or substituted bases. The protospacer adjacent motif (PAM) is highlighted in blue.
